# Label-Free Electrochemical Diagnosis of Viral Antigens with Genetically Engineered Fusion Protein

**DOI:** 10.3390/s120810097

**Published:** 2012-07-26

**Authors:** Nam Su Heo, Shun Zheng, MinHo Yang, Seok Jae Lee, Sang Yup Lee, Hwa-Jung Kim, Jung Youn Park, Chang-Soo Lee, Tae Jung Park

**Affiliations:** 1 BioProcess Engineering Research Center, KAIST, 291 Daehak-ro, Yuseong-gu, Daejeon 305-701, Korea; E-Mails: hns0924@kaist.ac.kr (N.S.H.); zshun@kaist.ac.kr (S.Z.); minhoyang@kaist.ac.kr (M.Y.); leesy@kaist.ac.kr (S.Y.L.); 2 Department of Chemical & Biological Engineering, Chungnam National University, 99 Daehangno, Yuseong-gu, Daejeon 305-764, Korea; 3 Center for Nanobio Integration & Convergence Engineering, National Nanofab Center, 291 Daehak-ro, Yuseong-gu, Daejeon 305-806, Korea; E-Mail: sjlee@nnfc.re.kr; 4 Department of Chemical & Biomolecular Engineering (BK21 Program), Department of Bio & Brain Engineering, Department of Biological Sciences, Bioinformatics Research Center, Center for Systems & Synthetic Biotechnology, and Institute for the BioCentury, KAIST, 291 Daehak-ro, Yuseong-gu, Daejeon 305-701, Korea; 5 Department of Microbiology & Research Institute for Medical Science, Chungnam National University, 6 Moonhwa-dong, Jung-gu, Daejeon 301-747, Korea; E-Mail: hjukim@cnu.ac.kr; 6 Biotechnology Research Division, National Fisheries Research & Development Institute (NFRDI), 408-1 Sirang-ri, Gijang, Busan 619-705, Korea; E-Mail: jypark@nfrdi.go.kr; 7 Department of Chemistry, Chung-Ang University, 84 Heukseok-ro, Dongjak-gu, Seoul 156-756, Korea

**Keywords:** hepatitis B virus, Gold-binding polypeptide, fusion protein, electrochemical analysis

## Abstract

We have developed a simple electrochemical biosensing strategy for the label-free diagnosis of hepatitis B virus (HBV) on a gold electrode surface. Gold-binding polypeptide (GBP) fused with single-chain antibody (ScFv) against HBV surface antigen (HBsAg), in forms of genetically engineered protein, was utilized. This GBP-ScFv fusion protein can directly bind onto the gold substrate with the strong binding affinity between the GBP and the gold surface, while the recognition site orients toward the sample for target binding at the same time. Furthermore, this one-step immobilization strategy greatly simplifies a fabrication process without any chemical modification as well as maintaining activity of biological recognition elements. This system allows specific immobilization of proteins and sensitive detection of targets, which were verified by surface plasmon resonance analysis and successfully applied to electrochemical cyclic voltammetry and impedance spectroscopy upto 0.14 ng/mL HBsAg.

## 1. Introduction

In recent years, various types of biosensors have been increasingly becoming practical and useful tools in a wide variety of analytical devices [1,2]. The immobilization of biological elements to realize the biosensor is an essential step for the successful construction of a diagnostic system. In order to allow the detection of a small amount of target sample and improve detection performance, bioreceptor proteins must be immobilized onto biosensor chip surfaces with high density and nonspecific adsorption avoided or at least minimized. Moreover, orientation control with retention of protein conformation and activity is a required task to be established [3]. One method involves the physical adsorption via van der Waals forces, ionic binding or hydrophobic and polar forces on an insoluble matrix. This is a simple process which causes little disruption of the proteins, while it is unstable during the binding procedure due to the highly dependency against environmental conditions in maintaining its functional characteristics. Thus, the resulting receptor layer seems to form heterogeneous and random orientation. Another method can also be constructed by crosslinking functional reagents by a certain number of functional groups due to its simple procedure and strong chemical bond of proteins. This is widely used for stabilization of proteins that are covalently bound onto the support platform generated by chemical treatment. However, this method has also disadvantages as follows: the difficulty in controlling the crosslinking reaction, the gelatinous nature of the proteins and the relatively low activity of the proteins due to the specific structural features [3,4]. In each of these methods used for the favorable performance of biosensors, a number of factors deserve careful consideration for strong binding forces between the solid surface and recognition elements, exposure of active sites on the target sample, conservation of biological activity after immobilization process, and simple protocol [3,4].

Many researchers have studied *in vivo* combinatorial biotechnology, e.g., either phage or cell-surface display techniques, and developed polypeptide sequences, which can specifically bind to metals [5–7], oxides [8,9], and semiconductors [10–12]. Among them, gold-binding polypeptide (GBP) is one of the genetically engineered proteins for a strong binding onto the gold surface [7,13,14]. Whereas many proteins and chemicals bind to the gold surface with thiol linkage, GBP does not contain a cysteine residue having thiol group. Although the definite mechanism is not clear yet, it is estimated that these polar groups in GBPs seem to coordinately interact with the gold surface within a monolayer [15,16]. In addition, the kinetics and thermodynamics of biomimetic interactions between the GBP, and the gold surface were investigated by surface plasmon resonance (SPR) [14,17,18]. Compared with other thiol-based systems, the GBP binds tightly to the gold surface due to the lower standard Gibbs free energy for the bond, and the binding process is fast under aqueous conditions compatible with biological environments [14,17,19]. These characteristics suggest its potential applications in nano- and bio-technologies as novel agents for surface functionalization [13].

**Figure 1.**
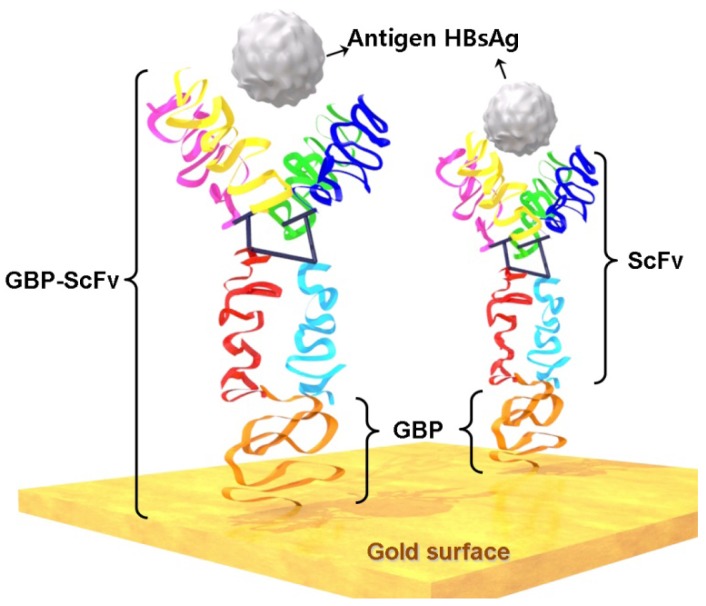
Schematic diagram of bio-recognition onto the gold surface by GBP-fusion proteins and detection of targets (Immobilization of single-chain variable fragment antibody via 6HGBP-ScFv fusion protein for the detection of HBsAg).

Moreover, immobilization with correct orientation of biological material is a problem of prime importance in biosensors. We employed GBP-fusion proteins in the construction of biosensor for the detection of hepatitis B viral surface antigen (HBsAg) as a model (Figure 1), which is a biomarker for diagnosing the hepatitis B virus (HBV). The strong affinity between the GBP and the gold surface guarantees the stability of this sensor system and orients the sensing parts outward from the solid surface, exposing them directly to the target sample [13,16]. Furthermore, electrochemical detection has attracted considerable interest recently for miniaturized analytical systems [20,21], including remarkable sensitivity (approaching that of fluorescence), inherent miniaturization of both the detector and control instrumentation, independence of optical path length or sample turbidity, low cost, low-power requirements and high compatibility [22,23]. Besides, one of the most attractive points of this method is its potential for portable assays in a variety of point-of-care testing (POCT) environments. We here developed a simple platform biosensor technology by mediating the recognition parts and the solid surface on the gold substrate. SPR analyses were used for optimization of sample concentrations and verification of target sensing. Electrical signal-based detection methods for HBV such as electrochemical impedance spectroscopy (EIS) and cyclic voltammetry (CV) were developed on the gold electrode surface, which has been a very versatile material in the field of biosensors.

## 2. Experimental Section

### 2.1. Reagents and Materials

Restriction enzymes were purchased from New England Biolabs (Beverly, MA, USA). Agarose was from Cambrex BioScience Rockland (Rockland, ME, USA). 30% (w/v) acrylamide/bis solution and protein assay were purchased from Bio-Rad (Hercules, CA, USA). HBsAg PreS2 peptide (H_2_N-NSTTFHQALLDPRVRGLYFPAGG-COOH) was synthesized at Peptron (Daejeon, Korea). Ni-NTA affinity kit was from Qiagen (Hilden, Germany). Other chemicals and reagents were purchased from Sigma-Aldrich (St. Louis, MO, USA), unless otherwise stated. All oligonucleotides were synthesized at Bioneer (Daejeon, Korea).

### 2.2. Apparatus

Polymerase chain reaction (PCR) experiments were performed with a PCR Thermal Cycler (Bio-Rad) using High-Fidelity PCR System (Boehringer Mannheim, Mannheim, Germany). DNA sequences were confirmed by automatic DNA sequencer (ABI Prism model 377, Perkin Elmer, Grove, IL, USA). Cell growth was monitored by measuring the absorbance at 600 nm (OD_600_; DU^®^650 spectrophotometer, Beckman, Fullerton, CA, USA). Cells were disrupted by using ultra-sonicator (Braun Ultrasonics, Danbury, CT, USA). SPR experiments were performed by using *SPRLAB*™ system (K-MAC, Daejeon, Korea) and BIAcore3000 (GE Healthcare, Uppsala, Sweden). Electrochemical detection analysis was carried out using CHI 660C Electrochemical Analyzer/Workstation (CH Instruments, Austin, TX, USA).

### 2.3. Production of 6HGBP-ScFv Fusion Protein

The flow-chart for the construction of the fusion protein between the GBP and its single-chain antibody (ScFv) is shown in Figure S1 (Supplementary material). The 6HGBP-ScFv fusion protein was prepared by genetically fusing the GBP and ScFv, allowing two specific interactions between GBP and gold substrates, and the capture of HBsAg and its ScFv, respectively. For easy purification of the fusion protein by metal affinity chromatography, the coding sequence of a six-histidine (6H) was introduced at the N-terminus of the GBP. For the cloning of the fusion gene, the DNA fragments encoding 6histidine-fused GBP (6HGBP) were obtained by PCR amplification using plasmid pTacFadLGBP-1 [16] as a template, and P1 (5′-AAAATACCATATGGGCCACCATCACCATCACCACGG-3′) and P2 (5′-TTCCCCATGGAGACGAATGGTACCGCTCGT-3′) as primers. The PCR product was digested with *Nde*I and *Nco*I, and ligated into the same sites of pET-22b(+) (Novagen, San Diego, CA, USA) to make pET-6HGBP. For the cloning and expression of the 6HGBP-ScFv fusion gene, the DNA sequence encoding ScFv fragment was amplified by PCR using plasmid pET-ScFv-SBD [18] as a template, and P3 (5′-CAAGACCATGGGTGTCGACTGAGGAGTCTGGA-3′) and P4 (5′-TCCGCTCGAGACGTTTTATTTCCAGGTAGGT-3′) as primers. This PCR product was digested with *Nco*I and *Xho*I, and ligated into the same sites of pET-6HGBP to make pET-6HGBP-ScFv.

*Escherichia coli* BL21(DE3) [F^-^ *ompT hsdS*_B_ (r_B_^-^ m_B_^-^) *gal dcm* (DE3)] was used as a host strain for the expression of GBP-fused ScFv fragment (GBP-ScFv). Recombinant *E. coli* BL21(DE3) strain harboring pET-6HGBP-ScFv was cultivated in 250 mL flasks containing 100 mL Luria-Bertani medium supplemented with 2% (w/v) glucose and 100 μg/mL of ampicillin at 37 °C in a rotary shaking incubator. Cell growth was monitored by measuring the absorbance at 600 nm. At an OD_600_ of 0.4, isopropyl-β-D-thiogalactopyranoside (IPTG) was added to a final concentration of 1 mM to induce the gene expression. Then, cells were further cultivated for 6 h and harvested by centrifugation. Cells were disrupted by sonication for 1 min at 40% output in cell lysis solution (Tris-NaCl buffer containing 6 M GuHCl and 5 mM imidazole, pH 8.0) and centrifuged at 16,000× g for 10 min at 4 °C. The pellet containing insoluble proteins was denatured, purified and underwent dialysis for further experiments. Since the 6HGBP-ScFv fusion protein contains 6H tag at N-terminal, they could be simply purified using Ni-chelating resin (Qiagen, Valencia, CA, USA) without further purification step. Protein concentration was determined by Bradford's method using bovine serum albumin (BSA) as a standard.

### 2.4. SPR Spectroscopy Analysis for HBV Diagnosis

The SPR experiments were carried out at 25 °C by using a repeated angle mode and a fixed angle mode. The repeated mode is the method for measuring the changes of the minimum resonance angle in specially fixed angle range by repetitive angle scanning and fitting, which is a plot of change in the reflectance intensity as a function of time (Resonance *vs.* Time). *SPRLAB*™ (K-MAC, Korea) system detects a refractive index and thickness change containing semiconductor laser source with 635 nm wavelength and dielectric silicon photodiode detector, which has incident angle range of 30–80 degree.

For the HBsAg detection, the concentration of 6HGBP-ScFv fusion protein was roughly optimized at first. Sequential injection of solutions was as follows: For equilibration, phosphate-buffered saline (PBS) solution was flowed over the bare gold surface to wash away any potential contaminants. Then, several different concentrations of 6HGBP-ScFv fusion protein were injected. The optimal concentration of 6HGBP-ScFv fusion protein, determined in this process, was used for detecting various concentrations of target HBsAg in the following experiments. In the process of HBsAg detection, BSA instead of the target was injected as a negative control. Prior to the target binding, 0.5 mg/mL of BSA was injected with a flow rate at 20 μL/min for an effective blocking of nonspecific binding sites. In order to remove nonspecifically bound molecules and unbound samples, washing step was applied intermittently with PBS for about 5 min. For the binding of targets, a fixed flow rate at 5 μL/min and binding time of 5 min was applied, consuming a total sample volume of 100 μL.

### 2.5. Electrochemical Measurement for HBV Diagnosis

Electrochemical measurements including EIS and CV were performed on a conventional electrochemical cell equipped with Ag/AgCl with 3 M KCl as a reference electrode, platinum wire as a counter electrode and bare gold electrode as a working electrode. All potentials were referred to the Ag/AgCl reference electrode.

Immediately prior to use, working electrodes were cleaned by five cycles of CV in a potential window of −0.5 to 0.5 V to remove any potential contaminants. Then, the clean gold surface was serially modified by sequential immersion in 50 μg/mL 6HGBP-ScFv and 0.5 mg/mL BSA as a negative control for 1 h at room temperature, respectively. In the last step, the modified electrodes were incubated with various concentrations of HBsAg solution for about 2 h. The washing step with DI water and nitrogen gas was performed after each binding event.

The electrode fabrication process, namely, confirmation of binding of recognition elements onto the gold electrode was characterized by EIS and CV, while EIS was used for target detection. Electrochemical measurements including EIS and CV were performed in 1 mM ferricyanide in 0.1 M KCl (Nyquist plot). CV experiments were carried out in unstirred solutions at a scan rate of 0.1 V/s and at a fixed potential window of −0.5 to 0.5 V *vs.* Ag/AgCl. Impedance measurements were carried out in the frequency range from 10^4^ down to 0.1 Hz with AC amplitude of 5 mV and bias potential of 0.22 V.

## 3. Results and Discussion

### 3.1. Preparation of 6HGBP-ScFv Fusion Protein

To verify the expression level of the 6HGBP-ScFv fusion proteins, the sodium dodecyl sulfate polyacrylamide gel electrophoresis (SDS-PAGE) of 6HGBP-ScFv fusion protein was carried out as shown in Figure S2 (Supplementary material). Compared with the wild-type *E. coli* BL21(DE3) strain as a negative control, *E. coli* BL21(DE3) harboring pET-6HGBP-ScFv expresses a thick band between marker bands of size 27.5 kDa and 37 kDa. This band size is in agreement with the size of 6HGBP-ScFv fusion protein, which was calculated as approximately 31.9 kDa. Since this fusion protein is expressed as an insoluble fraction, it was purified in denaturing condition and sequentially subjected to dialysis. According to the result, the most right band shows the 6HGBP-ScFv fusion protein purified using a Ni-chelating column resulting with good purity and quantity for further use in several sensing platforms.

### 3.2. HBV Diagnosis by SPR Analysis

SPR is a label-free and sensitive spectroscopic technique used to study bioaffinity interactions on gold thin films by measuring changes of the refractive index under conditions of total internal reflection [24,25]. To prove that GBP-fusion proteins could be functionally immobilized on the gold surface, the 6HGBP-ScFv fusion protein was flown over the gold sensor chip to monitor its binding affinity by combining the GBP-fusion approach with SPR. The dynamic and specific binding of fusion proteins onto the gold sensor chip could be directly monitored in real time by Biacore^®^ SPR spectroscopy (Figure S3 in Supplementary material). A sharp increase in the SPR signal was observed upon introducing the 6HGBP-ScFv fusion protein solution onto the chip surface. About 93% of the injected total 6HGBP-ScFv fusion proteins were bound onto the gold sensor chip surface. Most 6HGBP-ScFv proteins remained bound to the gold surface after washing with PBS solution. Whereas a little decline in SPR signal during washing is due to the removal of unbound 6HGBP-ScFv proteins from the gold surface, the SPR signal sharply decreased after washing with the PBS solution when ScFv instead of 6HGBP-ScFv was flown over the gold sensor chip. About 23% of ScFv protein was nonspecifically bound to the gold surface compared with 6HGBP-ScFv fusion protein. The binding affinities were calculated by assuming a single binding site model as shown in Table S1. Strong binding of GBP-fusion protein onto the gold chip surface suggests that various protein-protein interactions can be performed by using this system.

**Figure 2.**
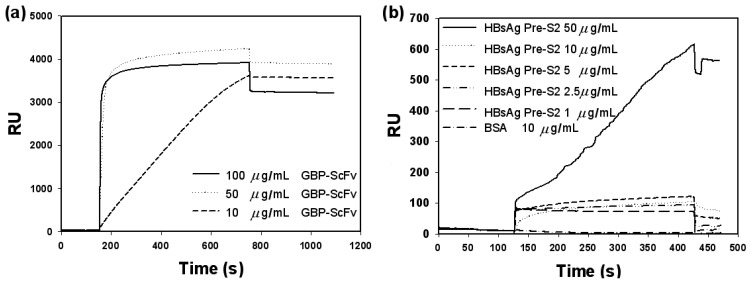
SPR sensorgrams. (**a**) Optimization of the concentration of 6HGBP-ScFv fusion protein. (**b**) SPR detection of target antigen, HBsAg, with 6HGBP-ScFv as a receptor.

To confirm the successful binding of a series of molecules, the detection of HBV was first performed using commercial SPR system. At first, 6HGBP-ScFv concentration was optimized as follows: 100, 50 and 10 μg/mL of 6HGBP-ScFv fusion protein was flowed over the gold surface, respectively. The fusion protein 6HGBP-ScFv was immobilized onto the gold surface via its intrinsic affinity with the gold as shown in Figure 2. Interestingly, coverage of 6HGBP-ScFv on the gold surface didn't always increase with increasing protein concentrations, a maximal RU change appearing at concentrations of 50 μg/mL. From this result, 6HGBP-ScFv concentration of 50 μg/mL was chosen for subsequent experiments.

Next, we checked the specificity of this platform with BSA as a negative control. First, 50 μg/mL of 6HGBP-ScFv was injected into the fluid phase, followed by 0.5 mg/mL BSA blocking of the binding sites on the fusion protein. Then, 10 μg/mL of control peptide (HOOC-CGPTGPTGPTGPTGPT-NH_2_) as a negative control, 50, 10, 5, 2.5 and 1 μg/mL of target HBsAg were injected into respective channels, respectively. Below concentrations of 50 μg/mL for HBsAg, RU levels dropped rapidly while target concentrations as low as 2.5 μg/mL can be detected compared with the control sample. For 50, 10, 5, 2.5 and 1 μg/mL of target HBsAg, and 0.5 mg/mL of BSA, RU increase is 542.2 RU, 55.7 RU, 48.5 RU, 17.4 RU, 4.9 RU and 5.6 RU, respectively, showing an increasing trend of SPR signals with increasing concentration of target within this scope.

### 3.3. HBV Diagnosis by Electrochemical Measurement

To detect HBsAg, electrochemical assay was also carried out, which have traditionally received the major share of the attention in biosensor development for their various advantages such as high sensitivity, specificity and simplicity, and inherent miniaturization of modern electrical bioassays permits them to rival the most advanced optical protocols [26]. Such miniaturization allows packing of numerous microscopic electrode transducers onto a small footprint of the biosensor device, and hence the design of high-density arrays was required. In this paper, EIS and CV were performed in order to characterize the electrode fabrication process, and EIS was applied to detect HBsAg. For the determination of optimal 6HGBP-ScFv coverage, its concentration and volume were carefully calculated in regard to that used in SPR analysis.

**Figure 3.**
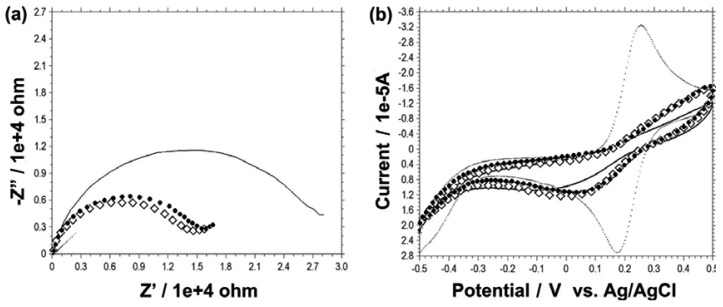
Electrochemical detection of HBsAg. (**a**) EIS characterization and (**b**) CV analysis of the gold electrode fabrication process for the sequential binding of the samples. Dotted line, bare gold; Frame diamond, after 50 μg/mL 6HGBP-ScFv binding; Solid circle, after 0.5 mg/mL BSA binding; Solid line, after 50 μg/mL HBsAg binding.

For the characterization of the electrode fabrication process, EIS and CV were performed each time after binding of each reagent. As shown in Figure 3(a), the dotted line is the impedance spectrum obtained on the bare gold electrode. Frame circle line is an impedance spectrum of 50 μg/mL 6HGBP-ScFv fusion protein modified electrode, and solid circle line is that of after BSA blocking. Finally, solid line represents an impedance spectrum obtained after HBsAg binding. The result reveals the resistance of the electron transfer at the electrode surface increasing step by step because of the insulating effect of the binding proteins. This electron transfer resistance at the interface on the electrode surface, and solution can be determined by the diameter of the semi-circle of the curves in EIS spectra. After the gold electrodes were immobilized with GBP-ScFv, the peak current decreased dramatically with an increase of the peak-to-peak potential separation (Δ*E*p) for the bare gold electrode. When HBsAg was bound on the gold chip surface, the peak current more decreased and Δ*E*p (approximately 110 mV) increased comparing with those of the gold electrode and the GBP-ScFv immobilized chip, resulting from the electron transfer resistance of bound HBsAg molecules. BSA was used as a negative control. It can also be observed that the current responses in CV spectra (Figure 3(b)) are decreasing in the process of electrode fabrication, which coincides with a conclusion drawn from impedance assay. These results were due to the insulating characteristics of the protein.

**Figure 4.**
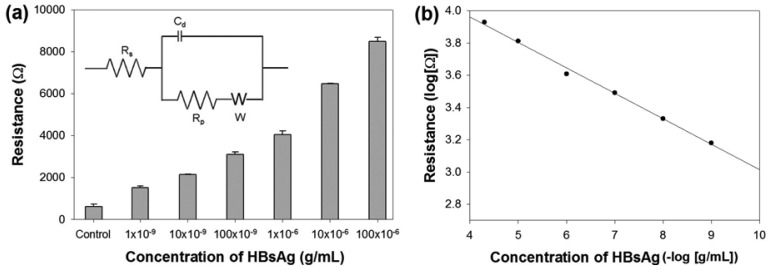
(**a**) EIS detection of different concentrations of HBsAg. 10 μg/mL, 1 μg/mL, 100 ng/mL, 10 ng/mL and 1 ng/mL of HBsAg were detected with 10 μg/mL BSA as a negative control, respectively. Numerical data was fitted with circuit as shown in Figure 3(a) (Inset: an equivalent circuit representing each component at the interface and in the solution during an electrochemical reaction is shown for comparison with the physical components. C_d_, double layer capacitor; R_p_, polarization resistor; W, Warburg resistor; R_s_, solution resistor). Error bars represent standard deviations from 5-time measurements. (**b**) Linear calibration curve into log scale of EIS data (*R*^2^ = 0.97).

As shown in Figure 4, 50 μg/mL, 10 μg/mL, 100 ng/mL, 10 ng/mL and 1 ng/mL of HBsAg were detected by using this method, respectively, and they presented a rough trend of increasing electron transfer resistance with increasing target concentrations. As a negative control, BSA of 10 μg/mL was detected at the same time. Numerical data were drawn by fitting with a circuit model as shown in an inset of Figure 4. Correspondingly, binding of 50 μg/mL, 10 μg/mL, 100 ng/mL, 10 ng/mL and 1 ng/mL of HBsAg and 10 μg/mL BSA caused electron transfer resistance increase of 8,493 Ω, 6,473 Ω, 4,047 Ω, 3,097 Ω, 2,143 Ω, 1,513 Ω and 777 Ω, respectively. Though linearity is not very strict as a function of HBsAg concentration, it demonstrated a rough linear trend into log scale. Target concentrations as low as 0.14 ng/mL of HBsAg calculated via 3-sigma rule was successfully detected compared with the negative control of 10 μg/mL of BSA (Figure 4). Furthermore, a lower limit of detection (LOD) can be further expected if experimental conditions, such as 6HGBP-ScFv concentration, binding time, temperature and washing condition, were optimized. In order to check the nonspecific binding of real blood sample, we tested a fetal bovine serum of 10 μg/mL instead of HBsAg for clinical trials [27,28] and confirmed no effect.

Several procedures for HBV diagnosis with GBP-fusion protein were developed in this study. First, 6HGBP-ScFv fusion protein was prepared from simple cultivation of recombinant *E. coli* for HBsAg detection. This GBP-fusion protein allowed for the direct and easy immobilization onto the gold surface. Successful binding of the proteins was demonstrated by SPR optical analysis due to versatile use of gold in the sensing area. Second, this one-step immobilization process onto the solid surface is very simple. Strong affinity between the GBP and the gold surface guarantees the stability preventing possible leakage of sensing proteins. Binding of GBP onto the gold surface also might help to expose sensing molecules outward to react with their targets by arrangement for correct orientation of bioreceptor. Additionally, GBP prevents direct contact between proteins and the gold surface, which is advantageous for protein activity conservation. Furthermore, it is easy to conclude that a device capable of detecting multiple targets can be designed since the bio-recognition elements against targets can be easily fused with GBP via recombinant DNA technology or linker chemistry. Coupling with microfluidics, it can serve to minimize sample volume required as well as to decrease diagnosis time.

## 4. Conclusions

In summary, we have developed a label-free electrochemical method for HBV detection based on a gold-specific immobilization strategy. The results showed that optimal concentration of bioreceptor was 50 μg/mL in SPR analysis, and the LOD for HBsAg was about 0.14 ng/mL in the electrochemical analysis. This EIS analysis successfully presents the effectiveness of this sensing platform. Regarding intrinsic advantageous property of the electrochemical assay, such as high sensitivity, simplicity and especially its inherent miniaturization characteristics, and a popular trend of development of electricity-based sensors in everyday life, the proposed method presented in this study has a huge potential in commercialization of a POCT device for viral diagnosis. We have to be conducted with clinical trials in the near future to determine its performance.

## Supplementary Material
