# Supplementary material for: Label-Free Electrochemical Diagnosis of Viral Antigens with Genetically Engineered Fusion Protein
Source: Sensors (Basel). 2012 Jul 26;12(8):10097–108. doi: 10.3390/s120810097 (PMC3472818; doi:10.3390/s120810097)
Supplement: Supplementary file 1 [file sensors-12-10097-s001.pdf]

OPEN ACCESS

sensors

ISSN 1424-8220

www.mdpi.com/journal/sensors

Supplementary Information

Label-Free Electrochemical Diagnosis of Viral Antigens with Genetically Engineered Fusion Protein. *Sensors* 2012, *12*, 10097-10108

Nam Su Heo [1,2,†], Shun Zheng [1,†], MinHo Yang [1], Seok Jae Lee [3], Sang Yup Lee [1,4], Hwa-Jung Kim [5], Jung Youn Park [6], Chang-Soo Lee [2,*] and Tae Jung Park [7,*]

[1] BioProcess Engineering Research Center, KAIST, 291 Daehak-ro, Yuseong-gu, Daejeon 305-701, Korea; E-Mails: hns0924@kaist.ac.kr (N.S.H.); zshun@kaist.ac.kr (S.Z.); minhoyang@kaist.ac.kr (M.Y.); leesy@kaist.ac.kr (S.Y.L.)

[2] Department of Chemical & Biological Engineering, Chungnam National University, 99 Daehangno, Yuseong-gu, Daejeon 305-764, Korea

[3] Center for Nanobio Integration & Convergence Engineering, National Nanofab Center, 291 Daehak-ro, Yuseong-gu, Daejeon 305-806, Korea; E-Mail: sjlee@nnfc.re.kr

[4] Department of Chemical & Biomolecular Engineering (BK21 Program), Department of Bio & Brain Engineering, Department of Biological Sciences, Bioinformatics Research Center, Center for Systems & Synthetic Biotechnology, and Institute for the BioCentury, KAIST, 291 Daehak-ro, Yuseong-gu, Daejeon 305-701, Korea

[5] Department of Microbiology & Research Institute for Medical Science, Chungnam National University, 6 Moonhwa-dong, Jung-gu, Daejeon 301-747, Korea; E-Mail: hjukim@cnu.ac.kr

[6] Biotechnology Research Division, National Fisheries Research & Development Institute (NFRDI), 408-1 Sirang-ri, Gijang, Busan 619-705, Korea; E-Mail: jypark@nfrdi.go.kr

[7] Department of Chemistry, Chung-Ang University, 84 Heukseok-ro, Dongjak-gu, Seoul 156-756, Korea

† Theses authors contributed equally to this work.

* Authors to whom correspondence should be addressed; E-Mails: rhadum@cnu.ac.kr (C.-S.L.); tjpark@cau.ac.kr (T.J.P.); Tel.: +82-2-820-5196; Fax: +82-2-825-4736.

Received: 10 May 2012; in revised form: 12 July 2012 / Accepted: 15 July 2012 / Published: 26 July 2012

SPR Analysis and Immobilization

The kinetic binding of GBP-ScFv fusion protein onto the surface of the SPR bare gold chip was characterized by SPR measurement using a BIAcore3000^{TM} instrument (Biacore AB, Uppsala, Sweden) with an automatic flow injection system. A fresh SPR sensor chip was attached to a separate chip carrier for easy assembly in the SPR system. After the SPR chip was docked and primed, phosphate-buffered saline (PBS) was used to flush the activated surface, to minimize non-specific binding and any unbound sites by removing loosely bound material and dust.

To examine successful immobilization of the genetically engineered fusion protein, GBP-ScFv, onto the gold sensor chip, 50 μL of the GBP-ScFv fusion protein (50 μg/mL) and the ScFv protein (50 μg/mL) as a negative control was bound onto the SPR gold chip for 10 min using a liquid-handling micropipette in the SPR system. Each surface was then washed with PBS. As shown in Figure S3, the dynamic and specific binding of GBP-ScFv onto the SPR chip was directly monitored in real-time. The SPR responses are indicated in resonance units (RUs). One RU corresponds to about 1 pg/mm of a bound biomolecule [1]. A sharp increase in the SPR signal up to about 4,000 RU was observed upon introducing GBP-ScFv solution onto the chip surface, and about 93% of the GBP-ScFv remained to the surface even after washing with PBS, which showed that most of the fusion protein was strongly immobilized onto the chip surface. The 4,000 RU value obtained implies that about 4 ng of GBP-ScFv was immobilized onto a gold surface area of 1 mm^2 [2]. All SPR experiments in this study were conducted in PBS at a flow rate of 5 μL/min at 25 °C, and all sensorgrams were fitted globally using BIA evaluation software.

Figure S1. Construction flow-chart of pET-6HGBP-ScFv expression vector in this study.

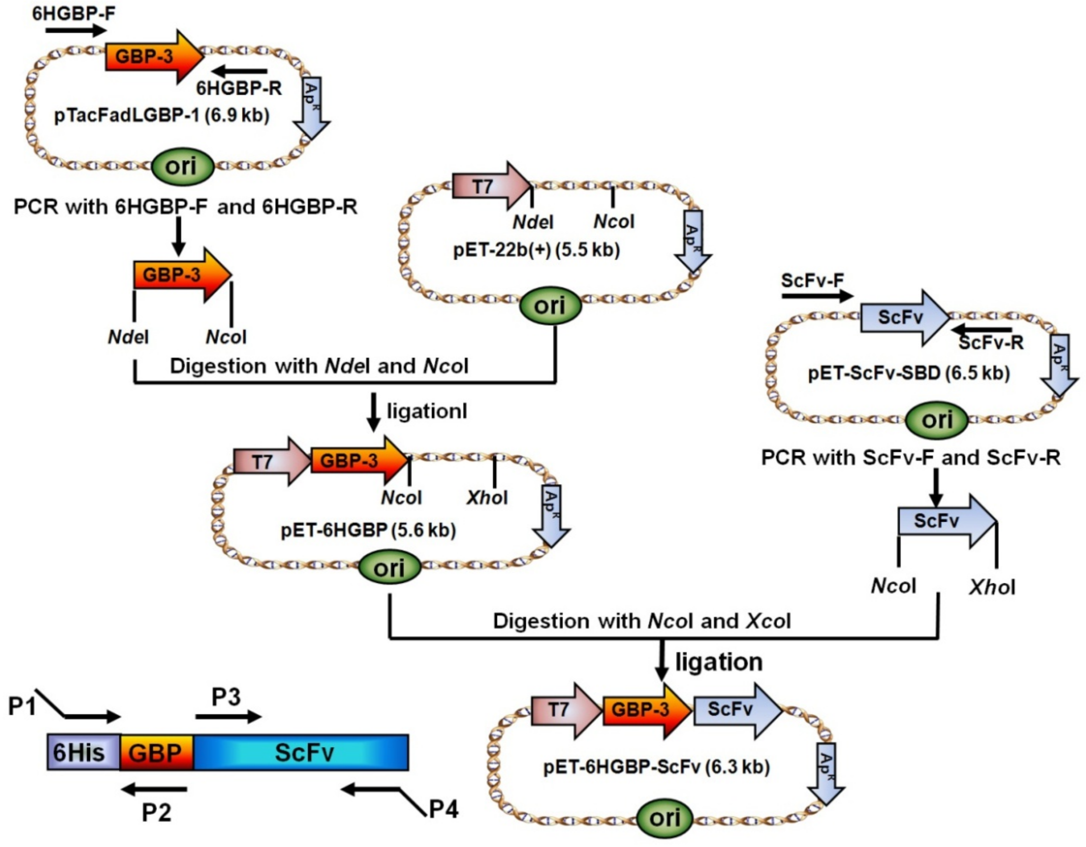

Figure S2. SDS-PAGE analysis of *E. coli* expressing 6HGBP-ScFv fusion protein (Arrow represents approximately 31.9 kDa). SM, protein size marker; 1, whole cell fraction of *E. coli* BL21(DE3) as a negative control; 2, whole cell fraction of *E. coli* BL21(DE3) expressing 6HGBP-ScFv; 3, soluble fraction of *E. coli* BL21(DE3) expressing 6HGBP-ScFv; 4, soluble fraction of *E. coli* BL21(DE3) expressing 6HGBP-ScFv; 5, purified 6HGBP-ScFv fusion protein.

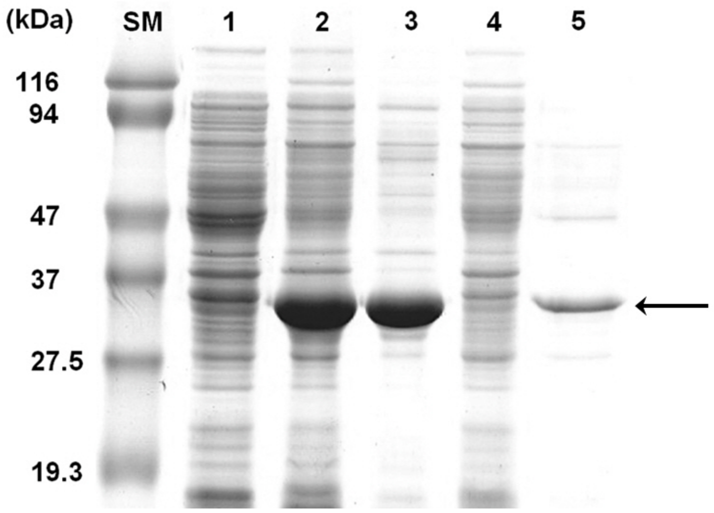

Figure S3. SPR sensorgrams showing the specific immobilization of 6HGBP-ScFv fusion proteins onto the gold sensor chip. The 6HGBP-ScFv fusion protein (dash line) and ScFv (boldface line) were introduced with 50 µg/mL at the flow rate of 5 µL/min. After protein binding, the surface of gold chip was washed with PBS solution.

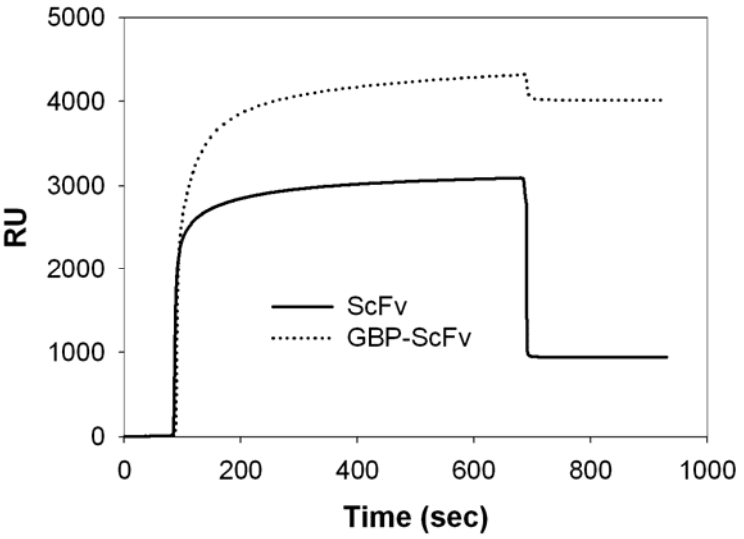

Table S1. The association rate constant (k_a), dissociation rate constant (k_d), and the equilibrium constant (K_D) of SPR data in Figure S3 were determined by BIAevaluation system *via* target proteins.

**Target protein (50 µg/mL)**	**k_a (1/M·s)**	**k_d (1/s)**	**R_{max} (RU)**	**k_a/k_d, K_A (1/M)**	**k_d/k_a, K_D (M)**
ScFv	2.54×10^{3}	1.09×10^{-6}	1.79×10^{4}	2.34×10^{9}	4.27×10^{-10}
6HGBP-ScFv	6.54×10^{3}	4.01×10^{-5}	1.94×10^{4}	1.63×10^{8}	6.13×10^{-9}

© 2012 by the authors; licensee MDPI, Basel, Switzerland. This article is an open access article distributed under the terms and conditions of the Creative Commons Attribution license (http://creativecommons.org/licenses/by/3.0/).
